# Differentiation of Gallbladder Adenomyomatosis and Polyps in a Western Cohort: Prevalence, Ultrasound Characteristics, and Diagnostic Challenges

**DOI:** 10.1002/jgh3.70343

**Published:** 2026-01-19

**Authors:** Marie Neumann, Michael Kallenbach, Ulrike Morgera, Andrea Cariati, Lars Morawietz, Frank Jacobsen, Frank Dubois, Sebastian Herberger, Falko Hanisch, Slim Khouja, Wolfram Wermke, Yvonne Dörffel

**Affiliations:** ^1^ Outpatient Clinic Charité – Universitätsmedizin Berlin Berlin Germany; ^2^ Clinic for Gastroenterology, Hepatology and Infectious Diseases Universitätsklinikum Düsseldorf Düsseldorf Germany; ^3^ General Surgery San Martino University and IST Hospital Genoa Italy; ^4^ Institute of Pathology Berlin‐Grunewald Berlin Germany; ^5^ Institute of Pathology, Universitätsklinikum Hamburg Eppendorf Hamburg Germany; ^6^ Institute of Pathology, Charité – Universitätsmedizin Berlin Berlin Germany; ^7^ Interdisciplinary Center of Sleep Medicine, Charité – Universitätsmedizin Berlin Berlin Germany

**Keywords:** conservative management, follow‐up studies, gallbladder adenomyomatosis, gallbladder polyps, ultrasonography

## Abstract

**Background:**

Gallbladder adenomyomatosis and polyps are common benign lesions that can mimic malignancy on imaging, often leading to unnecessary cholecystectomy. Despite frequent sonographic detection, the prevalence of adenomyomatosis in living cohorts remains poorly defined.

**Aim:**

To determine the prevalence and characterize the sonographic features of gallbladder adenomyomatosis and polyps in a large unselected cohort, and to assess clinical relevance.

**Methods:**

We retrospectively analyzed 2674 patients (≥ 16 years) who underwent abdominal ultrasound over 20 months. Examinations were performed by highly experienced sonographers using B‐mode, color Doppler, superb microvascular imaging and contrast‐enhanced ultrasound. Adenomyomatosis and polyps were classified based on morphology, wall involvement, vascularity, and Rokitansky–Aschoff sinuses (RAS). Follow‐up imaging was available in 68 of 123 patients with polyps (median 76 months).

**Results:**

Adenomyomatosis was diagnosed in 32 patients (1.2%). Characteristic features included hypoechoic or isoechoic thickened wall with anechoic or microlith‐filled RAS, often producing comet‐tail and twinkling artifacts. Only one patient required cholecystectomy due to symptomatic diffuse disease. Gallbladder polyps were identified in 123 patients (4.6%). Most polyps remained stable or showed minimal growth, with only three patients undergoing surgery, revealing two cholesterol polyps and one precancerous intracholecystic papillary neoplasm (0.04% of the total cohort).

**Conclusion:**

Structured, high‐quality ultrasound enables reliable differentiation of adenomyomatosis and benign polyps from lesions suspicious for malignancy. The vast majority of findings are benign, supporting conservative management. These results provide a reference standard for sonographic assessment and emphasize the importance of awareness and systematic evaluation of gallbladder wall abnormalities.

## 1 Introduction

The prevalence of gallbladder adenomyomatosis differs according to study setting: cholecystectomy series report rates of approximately 1%–9% [1], with higher values typically observed when the gallbladder is subjected to complete histopathological examination. Autopsy‐based studies describe lower frequencies of 1%–5% [2]. Although gallbladder adenomyomatosis is frequently detected by ultrasonography, no studies to date have systematically reported its prevalence based solely on ultrasound examinations in living patient cohorts.

The prevalence of gallbladder polyps shows an even broader range across populations and study designs. In population‐based ultrasound screening cohorts, reported prevalences range from 0.3% to 12.3% [3, 4]. For example, polyps were identified in 10 926 individuals (5.6%) undergoing screening ultrasound in Japan [5], while a large German cohort reported a prevalence of 6.1% [6].

Benign gallbladder lesions, such as adenomyomatosis and cholesterol polyps, frequently pose a diagnostic challenge in abdominal imaging. Imaging‐based suspicion of malignancy often leads to cholecystectomy, although incidental gallbladder carcinoma is rare, occurring in only 0.3%–0.89% of cases [7, 8]. The vast majority of resected specimens reveal benign histopathology. This underscores the importance of accurate preoperative diagnosis.

Adenomyomatosis is a benign condition characterized by hyperplastic changes of the gallbladder wall and Rokitansky–Aschoff sinuses (RAS). It generally requires no treatment unless accompanied by symptomatic cholelithiasis [9, 10]. Similarly, the majority of gallbladder polyps detected sonographically are benign, with cholesterol polyps representing the most common subtype [11, 12].

A major challenge in gallbladder lesion assessment is the overlap in sonographic features among different entities. Inexperience and incomplete use of available ultrasound criteria can lead to misdiagnosis and overtreatment [13]. Importantly, approximately one‐third of patients continue to experience symptoms after cholecystectomy, highlighting the importance of careful surgical indication [14, 15].

This study aims to close that gap by characterizing its sonographic features and differentiating them from polyps in a large cohort examined at a leading tertiary care center. The examinations were performed by highly experienced sonographers using state‐of‐the‐art ultrasound equipment, enhancing the reliability of our findings. Ultimately, we seek to improve noninvasive diagnostics and reduce unnecessary surgical interventions.

## 2 Methods

### 2.1 Ethical Approval

Ethical approval for this study was obtained from the Ethics Committee of Charité—Universitätsmedizin Berlin, Campus Virchow‐Klinikum (approval number EA2/041/19, date of approval: April 11, 2019).

### 2.2 Patient Selection

We retrospectively analyzed data from inpatients and outpatients (≥ 16 years) who underwent abdominal ultrasound during a 20‐month recruitment period. Eligibility required complete gallbladder documentation, including images, and no prior cholecystectomy. All patients fasted for at least 6 h before the examination. Ultrasound was performed for various clinical indications, not necessarily related to gallbladder pathology. Collected data included age, sex and gallbladder findings such as adenomyomatosis, polyps, wall changes, cholecystolithiasis, sludge, and cholecystitis.

Patients with gallbladder polyps were retrospectively followed to assess temporal changes in polyp size. Longitudinal ultrasound data were available for a subset of patients, with a median follow‐up of 76 months (range, 6–186 months).

### 2.3 Imaging and Analysis

Abdominal ultrasound was performed using Canon (Aplio i800, Canon Medical Systems GmbH) and Toshiba (Aplio i800, Aplio 500, Toshiba Medical Systems GmbH) systems. Examinations utilized the i8CX1 (1.8–6.2 MHz), 6C1 (1.5–6.0 MHz), or 11 L3 (3.0–8.0 MHz, linear) transducers. Image quality was optimized through field‐of‐view adjustments or zoom mode. In cases where it was unclear whether a polyp was attached to the gallbladder wall or disrupted mural layers, the linear 11 L3 transducer was additionally employed to improve visualization of wall stratification.

Color Doppler flow imaging (CDFI) was used to assess vascularity or detect twinkling artifacts. For indeterminate cases, superb microvascular imaging (SMI) and contrast‐enhanced ultrasound (CEUS) with SonoVue (1.2–1.6 mL, Bracco Imaging SpA, Milan, Italy) were applied to evaluate perfusion. Gallbladder adenomyomatosis was classified into diffuse, focal, and segmental types [16]. For gallbladder polyps, the number, size, localization, and sonomorphologic appearance were systematically recorded.

### 2.4 Examiner Expertise and Diagnostic Standard

All ultrasound examinations were performed by examiners with at least 10 years of experience (DEGUM levels I–III, German Society for Ultrasound in Medicine), each having conducted > 20 000 examinations. All pathological findings were independently reviewed by two senior examiners with over 30 years of experience and > 100 000 examinations. Typical signs of gallbladder adenomyomatosis confirmed by senior examiners served as the diagnostic reference standard. For gallbladder polyps, senior examiners ensured diagnostic consistency, particularly in differentiating likely benign from suspicious findings.

### 2.5 Statistical Analysis

Descriptive statistics were used to summarize patient demographics and gallbladder findings. Group differences were assessed using independent or Welch's *t*‐test, Pearson's chi‐square test, or Fisher's exact test, as appropriate. Statistical significance was defined as *p* < 0.05.

## 3 Results

### 3.1 Study Cohort

Abdominal ultrasound findings were collected from 3243 patients. Of these, 385 were excluded due to prior cholecystectomy and 184 due to incomplete gallbladder documentation, leaving 2674 patients for analysis. The cohort included 1181 men (44.2%) and 1493 women (55.8%), with a mean age of 55.6 ± 16.1 years (range: 16–96 years). Most patients had normal gallbladder findings (2223 patients, 83.1%). Among the 451 patients with gallbladder abnormalities, 276 (61.2%) had cholelithiasis or sludge without other gallbladder pathology, and 20 (4.4%) had gallbladder wall thickening due to identifiable causes (e.g., acute cholecystitis, hepatitis, hypoalbuminemia, ascites, heart or kidney failure, or portal hypertension). The remaining 155 patients (34.4%) presented with polypoid lesions or adenomyomatosis. The main sonographic and histopathologic features of gallbladder adenomyomatosis and polyps are summarized in Table 1.

**TABLE 1:** Ultrasound features differentiating gallbladder adenomyomatosis and benign gallbladder polyps.

	Adenomyomatosis	Benign gallbladder polyps
Prevalence	1.2% (32/2674)	4.6% (123/2674)
Type of wall thickening	Focal (fundal), segmental (hourglass) or diffuse (generalized) wall thickening	Sessile (hypoechoic interface) or pedunculated (stalk often not visible); largest lesion used as index
Echogenicity	Wall segments hypoechoic or isoechoic to liver	Polyp hyperechoic, distinct from wall; no invasion of liver
B‐mode	RAS (anechoic cysts ± microliths); clustered comet‐tail artifacts	Echogenic intraluminal lesion(s); solitary comet‐tail possible; smooth surface
Cholecystolithiasis	Associated with gallstones in 34.4%	Associated with gallstones in 10.6%
CDFI	Twinkling artifacts from microliths; no intramural vascularity	Usually no vascular signal
SMI	No microvascular signal within thickened wall	Absent or minimal punctate signals; single feeding vessel possible in polyps ≥ 9 mm
CEUS	Trilaminar wall enhancement; non‐enhancing intraluminal spaces (“moth‐eaten”)	Iso‐enhancing to wall if benign; early/heterogeneous enhancement and wall disruption → suspicious
Histologic findings	Epithelial hyperplasia with muscular hypertrophy and mucosal invaginations (RAS); RAS may contain bile, pigment or cholesterol microliths	Mostly cholesterol polyps; fibroepithelial (if present); precancerous ICPN (rare: 1 case in cohort)
Management/Clinical implication	Conservative if asymptomatic; surgery if symptomatic	Surveillance for < 10 mm and no risk features; surgery if significant growth, ≥ 10 mm, or suspicious features

Abbreviations: CDFI, color Doppler flow imaging; CEUS, contrast‐enhanced ultrasound; ICPN, intracholecystic papillary‐tubular neoplasm; RAS, Rokitansky–Aschoff sinuses; SMI, superb microvascular imaging.

### 3.2 Gallbladder Adenomyomatosis

**FIGURE 1:**
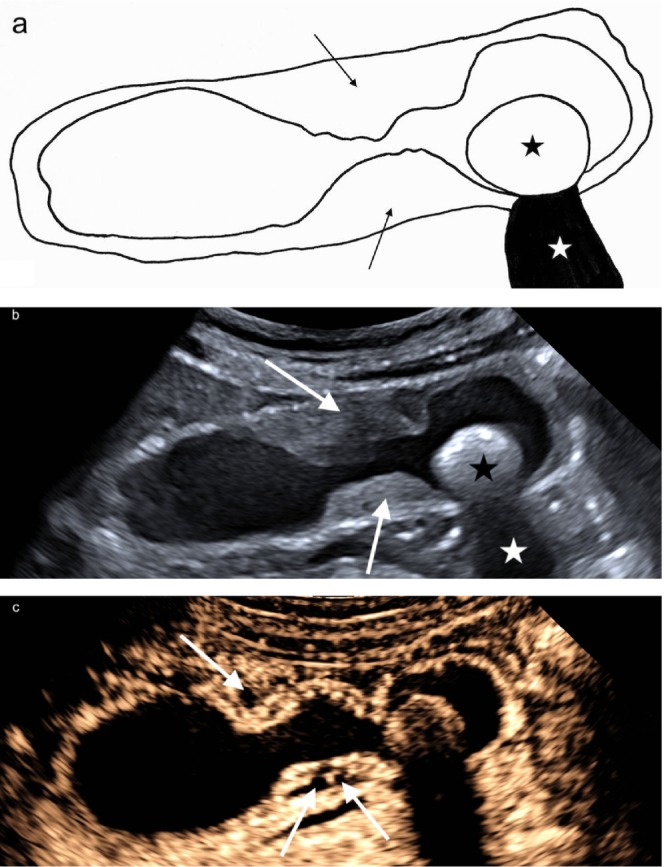
(a) Schematic representation of segmental gallbladder adenomyomatosis and cholecystolithiasis: Segmental wall thickening (black arrows) and gallstone (black star) with distal acoustic shadowing (white star). (b) Segmental wall thickening (white arrows) and gallstone (black star) with distal acoustic shadowing (white star) in B‐mode ultrasound. (c) Contrast‐enhanced ultrasound (CEUS) showing intact, homogeneously contrast‐enhanced gallbladder wall and non‐enhanced Rokitansky–Aschoff sinuses (RAS) (white arrows).

Adenomyomatosis was diagnosed in 32 patients (1.2%), consisting of 13 men (40.6%) and 19 women (59.4%) with a mean age of 59.5 ± 12.3 years (range: 32–88 years). Among these patients, 15 (46.9%) exhibited generalized involvement of the gallbladder wall (diffuse type), with the thickened wall often showing a “mountain chain–like” appearance. Fourteen patients (43.8%) had focal involvement (focal type), always affecting the fundus, which appeared hypoechoic and contained either anechoic cystic spaces and/or echogenic foci. Three patients (9.4%) presented with segmental involvement (segmental type), giving the gallbladder a characteristic “hourglass” appearance (Figure 1a–c).

**FIGURE 2:**
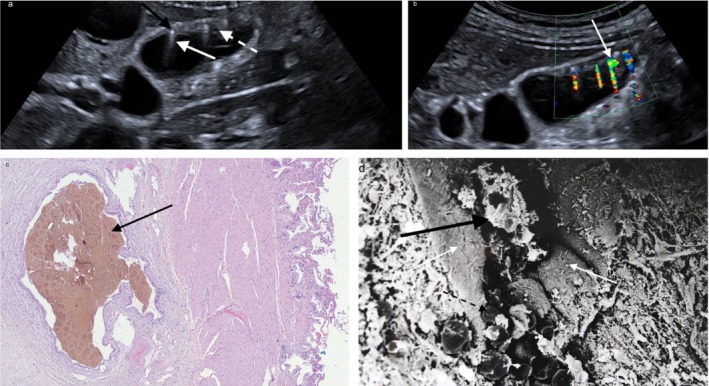
(a) Diffuse adenomyomatosis causing recurrent abdominal colics: Diffuse gallbladder wall thickening with bile‐filled RAS (white dashed arrow), microliths (black arrow), and comet‐tail artifacts (white arrow). (b) Twinkling artifacts (white arrow) on Color Doppler Flow Imaging (CDFI) (green CDFI ROI). (c) Histopathology showing dilated RAS with inspissated bile (black arrow) (Hematoxylin–eosin (HE); original magnification 5×). (d) Scanning electron microscopy (SEM, 0.199 kx) of a black pigment gallstone within a RAS. Cacodylate fixation caused epithelial alterations. Black pigment microstones (black arrow) are developing in RAS clefts (white arrows); in SEM they appear white and consist of calcium carbonate, mucin, and calcium bilirubinate. Early spherical calcium carbonate deposits without bilirubinate are visible in the fundus of the RAS (black dashed arrow).

The thickened wall segments were hypoechoic or isoechoic to the adjacent liver. RAS—intramural mucosal invaginations—were a consistent feature within the thickened wall and appeared either as small anechoic cystic spaces when bile‐filled or as punctate echogenic foci when containing microliths, the latter typically generating comet‐tail artifacts on B‐mode (Figure 2a) and twinkling on CDFI (Figure 2b). Across the cohort, RAS were bile‐filled only in 14 patients (43.8%), mixed bile‐filled and microlith‐containing in 16 (50.0%), and microlith‐only in 2 (6.3%). In cases with microliths, artifacts were usually multiple and clustered within the thickened wall segment (Figure 2b). On SMI, adenomyomatosis consistently showed absence of microvascular signals within the thickened gallbladder wall.

CEUS was performed in 4/32 patients (12.5%). In all cases, the gallbladder wall demonstrated preserved trilaminar enhancement in the arterial phase, with intramural non‐enhancing spaces corresponding to RAS, producing a characteristic “moth‐eaten” appearance, and no evidence of hyperenhancement or wall disruption (Figure 1c). In two patients, CEUS was performed incidentally during evaluation of adjacent liver lesions, while in the remaining two patients with segmental adenomyomatosis, CEUS was performed due to marked wall thickening.

Of the patients with adenomyomatosis, 18 (56.3%) had no other notable gallbladder findings, while 11 (34.4%) had concurrent cholecystolithiasis. Although patients with both adenomyomatosis and cholecystolithiasis were, on average, 4.1 years older than those with adenomyomatosis alone, this difference was not statistically significant (mean ± SD: 62.2 ± 12.3 vs. 58.1 ± 12.4 years; *p* = 0.381). Three patients (9.3%) had biliary sludge, considered a precursor to cholecystolithiasis.

Patients with adenomyomatosis had a significantly higher prevalence of gallstones (34.4%) than those with gallbladder polyps (10.6%). Statistical analysis confirmed this association (Chi‐square test: *p* = 0.0024; Fisher's exact test: *p* = 0.0022).

Among the patients with adenomyomatosis, all but one were asymptomatic. One patient (3.1%) required cholecystectomy due to recurrent upper abdominal colics. This patient had diffuse‐type adenomyomatosis with multiple small microliths within the RAS (Figure 2a). Histopathological examination confirmed the diagnosis of adenomyomatosis, and the microliths were identified as black pigment gallstones (Figure 2c,d).

### 3.3 Gallbladder Polyps

Gallbladder polyps were diagnosed in 123 patients (4.6%), comprising 61 men (49.6%) and 62 women (50.4%) with a mean age of 55.6 ± 14.1 years (range: 25–87 years). Among these patients, 110 (89.4%) had no other notable gallbladder findings. Patients with both gallbladder polyps and cholecystolithiasis were, on average, 15.1 years older than those with polyps alone, a difference that was highly significant (mean ± SD: 69.2 ± 10.7 vs. 54.0 ± 13.6 years; *p* < 0.0001).

Complete data on size, number, location, and morphology were available in 120/123 patients. Solitary polyps were present in 66 patients (55.0%), while 54 patients (45.0%) had multiple lesions (2 polyps in 32, 3 polyps in 16, and ≥ 4 polyps in 6 cases). The mean polyp size was 4.9 ± 2.0 mm (median 4.6 mm, range 1.6–17 mm). For patients with multiple polyps, the largest lesion was used for size analysis.

Overall, polyps were most frequently located in the corpus (*n* = 86), followed by the infundibulum (*n* = 41) and the fundus (*n* = 16). Because patients with multiple polyps could have lesions in more than one segment, numbers exceed the total cohort size.

Among the 66 patients with solitary polyps, 39 (59.1%) were located in the corpus, 20 (30.3%) in the infundibulum, and 7 (10.6%) in the fundus. Among the 54 patients with multiple polyps, the majority showed involvement of the corpus (*n* = 48, 88.9%). This included 26 patients with polyps confined to the corpus, 14 with corpus and infundibulum involvement, seven cases with corpus and fundus involvement, and a single case with all three segments affected. In contrast, only three patients (5.6%) had polyps confined to the infundibulum and two patients (3.7%) to the fundus.

**FIGURE 3:**
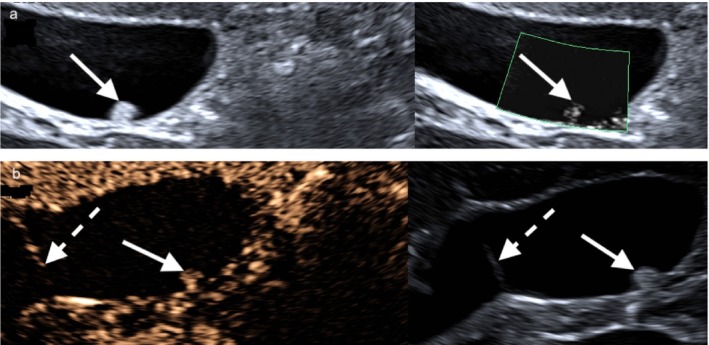
(a) Hyperechoic solitary polyp (white arrow) with smooth surface and no liver infiltration. Dotted vessels in the polyp with no prominent artery on Superb Microvascular Imaging (SMI) (green ROI). (b) Isoenhancing polyp (white arrow) measuring 8.7 mm, without late‐phase washout. A thin gallbladder septum is visible (dashed white arrow).

**FIGURE 4:**
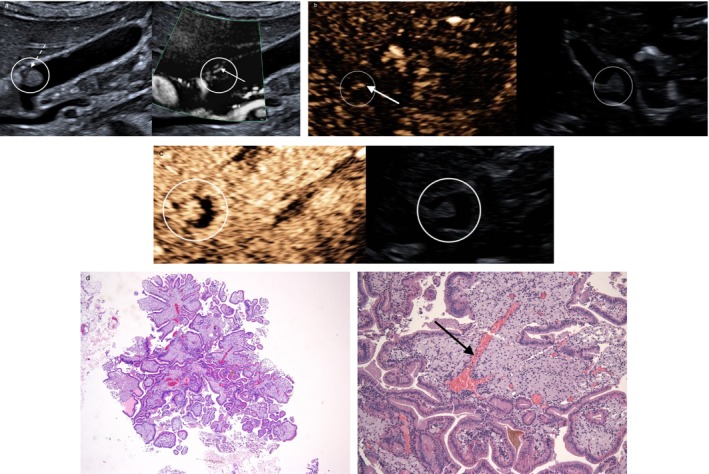
(a) Rapidly growing polyp (encircled in white) with disrupted gallbladder wall (white dashed arrow). It increased from 3 to 9 mm within a year. Prominent supplying artery (white arrow) on SMI (green ROI). (b) CEUS showing a straight supplying artery (white arrow) and regular blood flow in the solitary polyp (encircled in white). Corresponding B‐mode image showing the polyp with a smooth surface. (c) Cauliflower‐like enhancement of the polyp (encircled in white) on CEUS (arterial phase). Corresponding B‐mode image showing a smooth surface. (d) Histopathology showing a cauliflower‐like architecture of a cholesterol polyp (HE; original magnification ×20). The thin stalk detached during tissue fixation, which is a common artifact. (e) A vascular tree (black arrow) extending into the polyp (HE; high magnification 100×).

All polyps were hyperechoic. Morphologically, 52 lesions (43.3%) appeared pedunculated. In most cases, a thin stalk was not directly visualized. Instead, the polyps appeared as small, round lesions attached to the gallbladder wall, and distinction from a freely mobile gallstone was often only possible after patient repositioning. The remaining 68 lesions (56.7%) were sessile, typically demonstrating a hypoechoic interface between the lesion and the gallbladder wall, without evidence of invasion. In three cases of a sessile polyp, disruption of the wall layers suggested possible infiltration. Comet‐tail artifacts were rare, observed in only 6 of 120 patients (5.0%). Where present, they were always confined to the surface of a polyp rather than arising from multiple foci within a thickened wall segment. On SMI, gallbladder polyps either demonstrated no vascular signal or exhibited small punctate foci (Figure 3a), occasionally with a single feeding vessel in larger lesions (Figure 4a).

CEUS was performed in 5/120 patients (4.2%) with gallbladder polyps. In two cases, the indication was size progression during follow‐up. Both demonstrated isolated dot‐like vascularization confined to the polyp core without a feeding artery, wall disruption, or hyperenhancement (Figure 3b). In two patients with significant polyp growth, distinct patterns were observed: one showed incomplete visualization of the hypoechoic interface between the lesion and the gallbladder wall, while another exhibited both interruption of the wall layers (Figure 4a) and a feeding artery (Figure 4a,b). Both patients underwent cholecystectomy, and histopathology revealed cholesterol polyps (Figure 4d,e). The remaining patient underwent CEUS in the context of suspected liver metastases from colorectal carcinoma, with the gallbladder wall showing no abnormalities.

### 3.4 Follow‐Up of Gallbladder Polyps

Among the 123 patients with gallbladder polyps, 68 patients (55.3%) underwent follow‐up imaging, while 52 patients had no follow‐up. There were no significant differences in baseline characteristics (age, gender, or initial polyp size) between patients with and without follow‐up, suggesting minimal follow‐up bias. The mean number of follow‐up examinations per patient was 6.1 (median 5; range 2–18) over a mean follow‐up period of 75.4 months (median 76.4 months; range 6–186 months).

Polyp growth was categorized as follows: < 2 mm, stable or within measurement error; 2–4 mm, minimal growth; > 4 mm, significant growth. Index polyps were classified by initial size: small (< 5 mm), medium (5–9 mm), or large (> 9 mm). No large polyps were present initially.

Among small polyps, 37 remained stable, 12 showed minimal growth, and three demonstrated significant growth. Of these 3, two patients underwent cholecystectomy at their own request; histopathology confirmed cholesterol polyps. Maximal sizes at the time of surgery were 7 mm (growth from 2 mm over 40 months) and 9 mm (growth from 3 mm over 9 months). The remaining patient with significant growth opted for continued follow‐up, with the polyp enlarging from 2.8 to 7.8 mm over 53 months.

**FIGURE 5:**
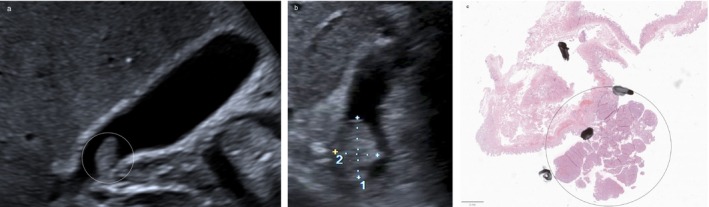
(a) Solitary 7 mm polyp‐like lesion with smooth surface (encircled in white). (b) Follow‐up after 30 months. The lesion increased to 17 mm in maximal diameter (measurement 1) and shows an irregular surface. (c) Polyp‐like lesion in the infundibulum with nodular surface. Histopathology reveals a tubular proliferation (encircled in black) consistent with an intracholecystic papillary‐tubular neoplasm (ICPN), pyloric‐type on immunohistochemistry.

Among medium‐sized polyps, 14 remained stable, one showed minimal growth, and one patient developed a polyp with significant growth, increasing from 7 to 17 mm over 30 months (Figure 5a,b). Cholecystectomy and histopathology revealed a completely excised intracholecystic papillary‐tubular neoplasm (ICPN) of the pyloric immunophenotype, without evidence of dysplasia or malignant transformation (Figure 5c).

Overall, 51 polyps remained stable, 13 exhibited minimal growth, four showed significant growth, and three patients underwent cholecystectomy during follow‐up.

## 4 Discussion

### 4.1 Prevalence of Gallbladder Adenomyomatosis and Polyps

The prevalence rates of adenomyomatosis and gallbladder polyps in our unselected cohort fall within the ranges reported in previous population‐based ultrasound studies [1, 2, 3, 4, 5, 6]. This suggests that our findings are adequately representative of routine abdominal imaging performed in clinical settings. However, the retrospective design introduces inherent limitations, including potential selection bias. For example, there is a possibility that subtle lesions may have been missed if documentation was incomplete or imaging windows were limited. At the same time, the retrospective inclusion of all consecutive abdominal ultrasound examinations—irrespective of indication—reduces referral bias and avoids the overrepresentation of symptomatic or preselected patients that commonly occurs in surgical or autopsy series. Thus, while not exempt from bias, our approach offers a complementary perspective to existing data derived from more selectively recruited populations.

### 4.2 Sonographic Characteristics and Diagnostic Implications

**FIGURE 6:**
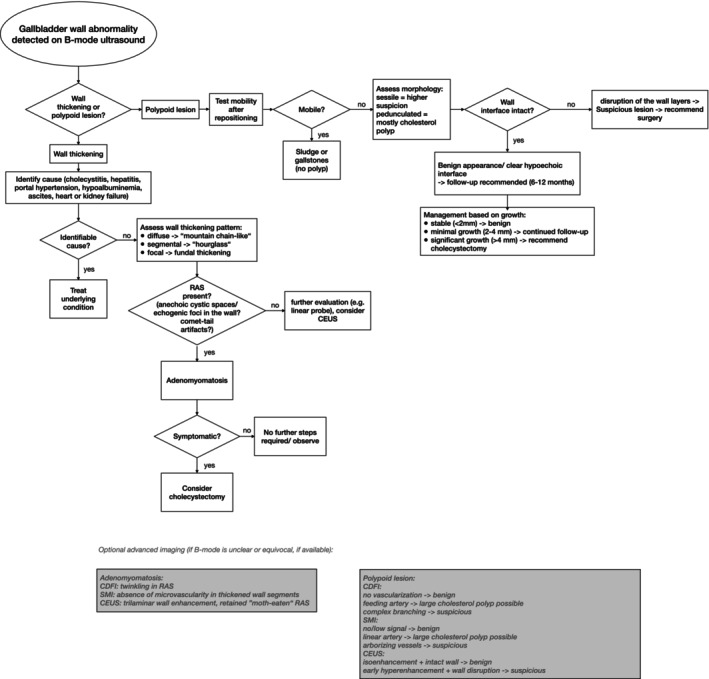
Stepwise B‐mode–based diagnostic algorithm for gallbladder wall abnormalities. The flowchart summarizes a structured ultrasound approach with primary emphasis on B‐mode criteria applicable in general hospital settings. After exclusion of gallstones, sludge, and secondary causes of wall thickening, lesions are categorized as adenomyomatosis or gallbladder polyps based on morphological features. Advanced imaging modalities (CDFI, SMI, CEUS) are indicated as optional adjuncts when B‐mode findings are inconclusive. Management recommendations are guided by lesion morphology and growth during follow‐up.

Abnormal gallbladder findings on ultrasound can be evaluated using a structured diagnostic pathway, as summarized in our flowchart (Figure 6). This tiered approach emphasizes B‐mode criteria applicable in general‐hospital settings, while advanced modalities such as CDFI, SMI, or CEUS act as optional adjuncts when B‐mode findings remain inconclusive.

Most abnormalities represent well‐characterized conditions such as gallstones or biliary sludge, which can be reliably identified using standard sonographic criteria, and typically do not require detailed wall assessment [17, 18, 19]. However, when gallbladder wall thickening is present, secondary causes such as inflammation, systemic disease, or vascular congestion must first be considered, underscoring the importance of integrating clinical context. After exclusion of these conditions, a smaller subset of patients with polypoid wall abnormalities—primarily adenomyomatosis or gallbladder polyps—which can be differentiated with more detailed morphological evaluation.

### 4.3 Gallbladder Adenomyomatosis

The sonographic features of adenomyomatosis observed in our cohort were highly consistent with established diagnostic criteria. RAS were identifiable in all patients and served as the key distinguishing feature, in line with prior studies emphasizing their central role in noninvasive diagnosis [20, 21]. Notably, in our cohort, RAS were already clearly detectable on B‐mode ultrasound in nearly all cases, indicating that reliable identification can be achieved even without advanced imaging modalities. CEUS may further increase diagnostic confidence by demonstrating the characteristic non‐enhancing intramural spaces [4, 11, 22, 23, 24].

### 4.4 Gallbladder Polyps

The sonographic features of gallbladder polyps in our cohort are consistent with established diagnostic patterns. Pedunculated, hyperechoic lesions without vascular complexity typically represent benign cholesterol polyps [25, 26, 27] whereas sessile morphology, loss of the hypoechoic interface, and documented size progression are the main features suggestive of neoplastic change [28, 29, 30, 31, 32]. Most polyps showed no meaningful growth during long‐term follow‐up (Figure S1), supporting conservative management in the absence of high‐risk features.

### 4.5 Clinical Outcomes and Management Implications

The clinical outcomes in our cohort were overwhelmingly benign. Only four patients showed significant polyp growth. Three underwent cholecystectomy, with histopathology revealing cholesterol polyps in two cases and a single ICPN without dysplasia in one case. These findings, together with the long median follow‐up of 76 months, reinforce current guideline recommendations that most small polyps can be safely monitored.

Although examinations were performed in a high‐expertise tertiary center, our findings remain applicable to routine practice. In all adenomyomatosis cases, the defining B‐mode features were identifiable on grayscale ultrasound alone. Among patients with polyps, CEUS was performed in only 5 of 123 cases and rarely altered interpretation, indicating that the essential diagnostic differentiation relied primarily on B‐mode characteristics.

A major limitation of this study is the lack of systematic histopathological confirmation. As only few patients underwent surgery, diagnoses were largely imaging‐based, and misclassification cannot be completely excluded. However, this limitation is partly mitigated by the clear B‐mode appearance of adenomyomatosis in all cases and by the long‐term stability of most polyps. The single ICPN identified emphasizes that neoplastic transformation, although rare, remains possible and supports continued surveillance of growing or atypical lesions.

Our analyses were primarily descriptive, focusing on prevalence and sonographic characteristics. Multivariable analyses of factors associated with polyp growth or gallstone development were beyond the scope of this study and were further precluded by the small number of events. Because only four patients exhibited significant growth, multivariable modeling would have been inappropriate due to sparse data and the risk of unstable or misleading estimates. Instead, we present an exploratory Kaplan–Meier analysis (Figure S1) of time to significant growth, which illustrates that clinically relevant progression was uncommon in our cohort.

## 5 Conclusion

Taken together, our findings demonstrate that most gallbladder wall abnormalities in an unselected ultrasound cohort are benign and can be safely managed conservatively.

Although examinations were performed in a high‐expertise setting, the essential diagnostic differentiation relied primarily on reproducible B‐mode criteria. To facilitate broader applicability, we provide a simplified stepwise B‐mode–based algorithm that supports structured assessment in general hospitals. Larger multicenter studies with systematic histopathology are needed to further validate this approach.

## Funding

The authors have nothing to report.

## Conflicts of Interest

The authors declare no conflicts of interest.

## Supporting information


**Figure S1:** Kaplan–Meier curve of gallbladder polyp stability. Kaplan–Meier curve illustrating the probability of no significant polyp growth (> 4 mm) during follow‐up. The analysis includes 68 patients with gallbladder polyps and a median follow‐up duration of 76 months. Only four events of significant growth occurred over the observation period, indicating that the majority of polyps remained stable during long‐term follow‐up.

## Data Availability

The data that support the findings of this study are available on request from the corresponding author. The data are not publicly available due to privacy or ethical restrictions.

## References

[1] N. Golse , M. Lewin , A. Rode , M. Sebagh , and J. Y. Mabrut , “Gallbladder Adenomyomatosis: Diagnosis and Management,” Journal of Visceral Surgery 154, no. 5 (2017): 345–353. 28844704 10.1016/j.jviscsurg.2017.06.004

[2] K. F. Lee , E. H. Y. Hung , H. H. W. Leung , and P. B. S. Lai , “A Narrative Review of Gallbladder Adenomyomatosis: What We Need to Know,” Annals of Translational Medicine 8, no. 23 (2020): 1600. 33437799 10.21037/atm-20-4897 PMC7791251

[3] M. Elmasry , D. Lindop , D. F. Dunne , H. Malik , G. J. Poston , and S. W. Fenwick , “The Risk of Malignancy in Ultrasound Detected Gallbladder Polyps: A Systematic Review,” International Journal of Surgery 33, no. Pt A (2016): 28–35. 27465099 10.1016/j.ijsu.2016.07.061

[4] Z. C. Riddell , C. Corallo , R. Albazaz , and K. G. Foley , “Gallbladder Polyps and Adenomyomatosis,” British Journal of Radiology 96, no. 1142 (2022): 20220115. 35731858 10.1259/bjr.20220115 PMC9975534

[5] M. Okamoto , H. Okamoto , F. Kitahara , et al., “Ultrasonographic Evidence of Association of Polyps and Stones With Gallbladder Cancer,” American Journal of Gastroenterology 94, no. 2 (1999): 446–450. 10022644 10.1111/j.1572-0241.1999.875_d.x

[6] W. Kratzer , A. Schmid , A. S. Akinli , et al., “Gallbladder Polyps: Prevalence and Risk Factors,” Ultraschall in der Medizin 32, no. Suppl 1 (2011): S68–S73. 20414857 10.1055/s-0029-1245265

[7] K. Soreide , R. V. Guest , E. M. Harrison , T. J. Kendall , O. J. Garden , and S. J. Wigmore , “Systematic Review of Management of Incidental Gallbladder Cancer After Cholecystectomy,” British Journal of Surgery 106, no. 1 (2019): 32–45. 30582640 10.1002/bjs.11035

[8] M. Altiok , H. G. Ozdemir , F. Kurt , M. O. Gul , and S. Gumus , “Incidental Gallbladder Cancer: A Retrospective Clinical Study of 40 Cases,” Annals of Surgical Treatment and Research 102, no. 4 (2022): 185–192. 35475225 10.4174/astr.2022.102.4.185 PMC9010968

[9] M. Bonatti , N. Vezzali , F. Lombardo , et al., “Gallbladder Adenomyomatosis: Imaging Findings, Tricks and Pitfalls,” Insights Into Imaging 8, no. 2 (2017): 243–253. 28127678 10.1007/s13244-017-0544-7 PMC5359147

[10] L. Pang , Y. Zhang , Y. Wang , and J. Kong , “Pathogenesis of Gallbladder Adenomyomatosis and Its Relationship With Early‐Stage Gallbladder Carcinoma: An Overview,” Brazilian Journal of Medical and Biological Research 51, no. 6 (2018): e7411. 29791592 10.1590/1414-431X20187411 PMC6002143

[11] M. H. Yu , Y. J. Kim , H. S. Park , and S. I. Jung , “Benign Gallbladder Diseases: Imaging Techniques and Tips for Differentiating With Malignant Gallbladder Diseases,” World Journal of Gastroenterology 26, no. 22 (2020): 2967–2986. 32587442 10.3748/wjg.v26.i22.2967 PMC7304100

[12] A. Kamaya , C. Fung , J. L. Szpakowski , et al., “Management of Incidentally Detected Gallbladder Polyps: Society of Radiologists in Ultrasound Consensus Conference Recommendations,” Radiology 305, no. 2 (2022): 277–289. 35787200 10.1148/radiol.213079

[13] E. Kim , R. Bashir , and S. Lahham , “Point‐Of‐Care Ultrasound Diagnosis of Cholecystitis vs. Adenomyomatosis,” Clinical Practice and Cases in Emergency Medicine 3, no. 2 (2019): 158–159. 31061976 10.5811/cpcem.2019.2.40798 PMC6497195

[14] D. M. Shabanzadeh , “The Symptomatic Outcomes of Cholecystectomy for Gallstones,” Journal of Clinical Medicine 12, no. 5 (2023): 1897. 36902684 10.3390/jcm12051897 PMC10004100

[15] F. M. Thunnissen , C. Baars , R. Arts , et al., “Persistent and New‐Onset Symptoms After Cholecystectomy in Patients With Uncomplicated Symptomatic Cholecystolithiasis: A Post Hoc Analysis of 2 Prospective Clinical Trials,” Surgery 174, no. 4 (2023): 781–786. 37541808 10.1016/j.surg.2023.06.010

[16] T. Ootani , Y. Shirai , K. Tsukada , and T. Muto , “Relationship Between Gallbladder Carcinoma and the Segmental Type of Adenomyomatosis of the Gallbladder,” Cancer 69, no. 11 (1992): 2647–2652. 1571894 10.1002/1097-0142(19920601)69:11<2647::aid-cncr2820691105>3.0.co;2-0

[17] L. I. Good , S. L. Edell , R. D. Soloway , B. W. Trotman , C. Mulhern , and P. A. Arger , “Ultrasonic Properties of Gallstones. Effect of Stone Size and Composition,” Gastroenterology 77, no. 2 (1979): 258–263. 447040

[18] C. Jungst , G. A. Kullak‐Ublick , and D. Jungst , “Gallstone Disease: Microlithiasis and Sludge,” Best Practice & Research. Clinical Gastroenterology 20, no. 6 (2006): 1053–1062. 17127187 10.1016/j.bpg.2006.03.007

[19] M. Zorniak , S. Sirtl , G. Beyer , et al., “Consensus Definition of Sludge and Microlithiasis as a Possible Cause of Pancreatitis,” Gut 72, no. 10 (2023): 1919–1926. 37072178 10.1136/gutjnl-2022-327955 PMC10511955

[20] P. J. Mariani and A. Hsue , “Adenomyomatosis of the Gallbladder: The “Good Omen” Comet,” Journal of Emergency Medicine 40, no. 4 (2011): 415–418. 19879088 10.1016/j.jemermed.2009.08.029

[21] A. Y. Hammad , J. T. Miura , K. K. Turaga , F. M. Johnston , M. D. Hohenwalter , and T. C. Gamblin , “A Literature Review of Radiological Findings to Guide the Diagnosis of Gallbladder Adenomyomatosis,” HPB 18, no. 2 (2016): 129–135. 26902131 10.1016/j.hpb.2015.09.006 PMC4814619

[22] S. Tang , L. Huang , Y. Wang , and Y. Wang , “Contrast‐Enhanced Ultrasonography Diagnosis of Fundal Localized Type of Gallbladder Adenomyomatosis,” BMC Gastroenterology 15 (2015): 99. 26239485 10.1186/s12876-015-0326-y PMC4524444

[23] J. F. Gerstenmaier , K. N. Hoang , and R. N. Gibson , “Contrast‐Enhanced Ultrasound in Gallbladder Disease: A Pictorial Review,” Abdominal Radiology 41, no. 8 (2016): 1640–1652. 27056746 10.1007/s00261-016-0729-4

[24] H. P. Zhang , M. Bai , J. Y. Gu , Y. Q. He , X. H. Qiao , and L. F. Du , “Value of Contrast‐Enhanced Ultrasound in the Differential Diagnosis of Gallbladder Lesion,” World Journal of Gastroenterology 24, no. 6 (2018): 744–751. 29456413 10.3748/wjg.v24.i6.744 PMC5807677

[25] A. Cariati and F. Cetta , “Rokitansky‐Aschoff Sinuses of the Gallbladder Are Associated With Black Pigment Gallstone Formation: A Scanning Electron Microscopy Study,” Ultrastructural Pathology 27, no. 4 (2003): 265–270. 12907372 10.1080/01913120309913

[26] V. M. Mellnick , C. O. Menias , K. Sandrasegaran , et al., “Polypoid Lesions of the Gallbladder: Disease Spectrum With Pathologic Correlation,” Radiographics 35, no. 2 (2015): 387–399. 25763724 10.1148/rg.352140095

[27] A. Xu , Y. Zhang , H. Hu , G. Zhao , J. Cai , and A. Huang , “Gallbladder Polypoid‐Lesions: What Are They and How Should They Be Treated? A Single‐Center Experience Based on 1446 Cholecystectomy Patients,” Journal of Gastrointestinal Surgery 21, no. 11 (2017): 1804–1812. 28695432 10.1007/s11605-017-3476-0

[28] O. C. Taskin , E. Bellolio , N. Dursun , et al., “Non‐Neoplastic Polyps of the Gallbladder: A Clinicopathologic Analysis of 447 Cases,” American Journal of Surgical Pathology 44, no. 4 (2020): 467–476. 31725469 10.1097/PAS.0000000000001405 PMC8693758

[29] X. S. Liu , L. H. Gu , J. Du , et al., “Differential Diagnosis of Polypoid Lesions of the Gallbladder Using Contrast‐Enhanced Sonography,” Journal of Ultrasound in Medicine 34, no. 6 (2015): 1061–1069. 26014326 10.7863/ultra.34.6.1061

[30] X. Wang , J. A. Zhu , Y. J. Liu , et al., “Conventional Ultrasound Combined With Contrast‐Enhanced Ultrasound in Differential Diagnosis of Gallbladder Cholesterol and Adenomatous Polyps (1‐2 Cm),” Journal of Ultrasound in Medicine 41, no. 3 (2022): 617–626. 33938029 10.1002/jum.15740

[31] L. Zhu , P. Han , B. Jiang , et al., “Value of Conventional Ultrasound‐Based Scoring System in Distinguishing Adenomatous Polyps From Cholesterol Polyps,” Journal of Clinical Gastroenterology 56, no. 10 (2022): 895–901. 34907919 10.1097/MCG.0000000000001639

[32] L. Zhu , P. Han , B. Jiang , Y. Zhu , N. Li , and X. Fei , “Value of Micro Flow Imaging in the Prediction of Adenomatous Polyps,” Ultrasound in Medicine & Biology 49, no. 7 (2023): 1586–1594. 37012096 10.1016/j.ultrasmedbio.2023.03.004

