# Supplementary figures and images for: Differentiation of Gallbladder Adenomyomatosis and Polyps in a Western Cohort: Prevalence, Ultrasound Characteristics, and Diagnostic Challenges
Source: JGH Open. 2026 Jan 19;10(1):e70343. doi: 10.1002/jgh3.70343 (PMC12816157; doi:10.1002/jgh3.70343)

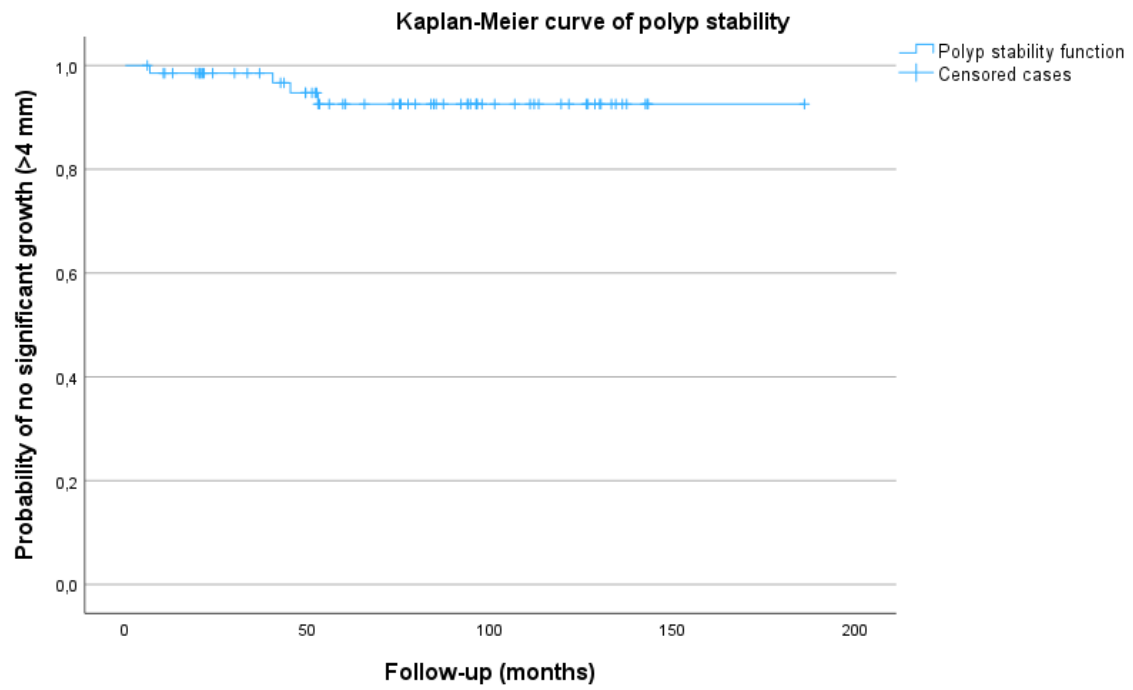

Supplement: Supplementary file 1 — Figure S1: Kaplan–Meier curve of gallbladder polyp stability. Kaplan–Meier curve illustrating the probability of no significant polyp growth (> 4 mm) during follow‐up. The analysis includes 68 patients with gallbladder polyps and a median follow‐up duration of 76 months. Only four events of significant growth occurred over the observation period, indicating that the majority of polyps remained stable during long‐term follow‐up. [file JGH3-10-e70343-s001.pdf]
